# Acute and long-term effects of repetitive transcranial magnetic stimulation in major depressive episodes: a systematic review and dose-response meta-analysis of randomized sham-controlled trials

**DOI:** 10.21203/rs.3.rs-8648926/v1

**Published:** 2026-02-02

**Authors:** Zuxing Wang, Ruanmei Sheng, Ruifeng Shi, Zhili Zou, Vaughn R. Steele, Xiaoyun Guo, Tifei Yuan

**Affiliations:** Shanghai Jiao Tong University; Shanghai Jiao Tong University; University of Electronic Science and Technology of China; University of Electronic Science and Technology of China; Yale University; Shanghai Jiao Tong University; Shanghai Jiao Tong University

**Keywords:** Transcranial magnetic stimulation, Major depressive episodes, Acute efficacy, Follow-up, Dose-response relationship

## Abstract

**Background:**

To characterize the dose-response relationships between key repetitive transcranial magnetic stimulation (rTMS) dosing parameters and clinical outcomes in major depressive episodes (MDE), and to identify optimal dosing ranges.

**Methods:**

We performed a systematic review and one-stage dose-response meta-analysis of randomized sham-controlled trials investigating rTMS for adults with MDE. Outcomes were symptom severity, response, and remission, assessed acutely and in follow-up (> 7 days). Four key dosing dimensions (total pulses, pulses/session, sessions, duration) were modeled using restricted cubic spline models, and the maximum effective dose (EDmax) within the observed range was derived from the fitted model. Effect sizes were expressed as standardized mean differences and risk ratios with 95% confidence interval.

**Results:**

Across 108 trials (134 active arms; n = 5,621), total pulses, pulses per session, total sessions and treatment duration showed significant non-linear associations with acute efficacy. Peak efficacy for acute treatment was observed at 30,000–39,000 total pulses, 1,800–2,200 pulses per session, 14–16 sessions, and 2.7–3.1 weeks of treatment, with no additional benefit from higher doses across all parameters. Long-term analyses showed smaller and mostly linear associations: the best outcomes were generally observed at 26,000–33,000 total pulses, 1,300–1,800 pulses per session, 10–14 sessions, and 2.8–3.3 weeks of treatment. Sensitivity analyses confirmed the stability of these estimates, and publication bias was minimal.

**Conclusions:**

rTMS efficacy in MDE is maximized within a moderate dose range, beyond which additional stimulation yields minimal gain. Thus, sustained remission likely depends on maintenance strategies, not dose escalation.

## Introduction

Major depressive episode (MDE) remains a leading cause of disability worldwide and imposes substantial clinical and economic burden [1]. Transcranial magnetic stimulation (TMS) is an established, guideline-endorsed, noninvasive treatment for depression [2]. However, clinical protocols vary widely in stimulation targets and dosing parameters (total pulses, pulses per session, number of sessions, and overall treatment duration), resulting in considerable heterogeneity in reported efficacy [3–6].

Recent dose-response syntheses point in complementary directions while leaving practical gaps [7–9]. In treatment-resistant depression (TRD), a multivariable meta-analysis reported that intensity, frequency, pulses per session, treatment duration, number of sessions, and total pulses explained meaningful variance [7]. Similarly, in TRD, a total-pulse-based analysis suggested a non-linear relationship peaking near 26,660 pulses, with frequency and age as significant moderators [8]. A trajectory-focused analysis showed a logarithmic improvement pattern with a plateau around weeks 3–4 and larger cumulative benefit at ≥3000 pulses per session and higher total pulses [9]. Additionally, a cross-protocol meta-analysis estimated near-maximal effective doses (ED95) and identified bell-shaped or ascending dose-response curves across paradigms using end-of-treatment assessments, across multiple psychiatric disorders [10].

Despite these advances, decision-relevant evidence remains limited. Previous studies have rarely examined the four dose dimensions together (total pulses, pulses per session, number of sessions, and treatment duration) or quantified their independent contributions while linking dosing to both acute and follow-up outcomes across continuous scores and binary endpoints (response and remission) in MDE. To address these gaps, we conducted a dose-response meta-analysis of major depressive episodes to examine the nonlinear associations of total pulses, pulses per session, number of sessions, and treatment duration with acute and follow-up outcomes, including depressive-symptom severity, treatment response, and remission.

## Methods

This systematic review and meta-analysis followed the Preferred Reporting Items for Systematic Reviews and Meta-Analyses (PRISMA) statement [11], and was prospectively registered in PROSPERO (registration number CRD420251051992).

### Search strategy

We systematically searched CENTRAL (Cochrane Central Register of Controlled Trials), Embase, and PubMed from inception to May 1, 2025, for randomized controlled trials (RCTs) of TMS in MDE, restricted to English-language publications. Reference lists of relevant reviews and articles were screened, and corresponding authors were contacted for missing data. Two reviewers independently screened titles, abstracts, and full texts in duplicate, resolving disagreements by discussion or a third reviewer. Complete search strategies are provided in the Supplement.

### Eligibility criteria

#### Participants (P).

Adults aged 18–65 years with a MDE in MDD or bipolar depression, diagnosed by DSM Diagnostic and Statistical Manual of Mental Disorders (DSM) [12] or International Classification of Diseases (ICD) [13]. Exclusion criteria included age < 18 or > 65, secondary mood disorders, severe cognitive impairment, or relapse-prevention/maintenance designs.

#### Interventions (I).

Therapeutic TMS at any cortical site, frequency, or coil type. Eligible modalities included repetitive TMS (high-frequency, low-frequency, bilateral, accelerated), theta-burst stimulation (TBS: intermittent [iTBS] and continuous [cTBS]), and deep TMS. Trials in which TMS was not the primary intervention were excluded.

#### Comparators (C).

Sham TMS using accepted inert methods (e.g., sham coil or angled coil). For cross-over trials, only pre-cross-over data were used.

#### Outcomes (O).

The six prespecified primary outcomes were assessed at two time points: an acute phase (end of treatment or within 7 days) and a follow-up phase (more than 7 days after treatment). Each window included three endpoints: continuous depressive-symptom severity, treatment response, and remission. Symptom severity was preferentially extracted from Hamilton Rating Scale for Depression (HAMD) [14], Montgomery-Asberg Depression Rating Scale (MADRS) [15], or Beck Depression Inventory (BDI) [16]. Symptom severity was extracted according to a prespecified hierarchy: HAMD first, then MADRS, and BDI if neither clinician-rated scale was available. Binary endpoints (response and remission) were extracted as reported in each trial, adhering to the trial’s prespecified definitions and analyses. We did not derive response or remission from continuous data when not reported. Where provided, common criteria included ≥ 50% reduction from baseline for response and instrument-specific remission cut-offs (e.g., HAMD-17 ≤ 7, MADRS ≤ 10).

#### Study design (S).

Peer-reviewed RCTs comparing active versus sham TMS, with extractable data.

### Data extraction

Two reviewers independently extracted trial-level data using a piloted form, with disagreements resolved by a third reviewer. For each study arm, we recorded author, year, and country; intervention details (active TMS parameters and sham control method); diagnosis (major depressive episode in MDD or bipolar depression) and diagnostic criteria (DSM or ICD); treatment-resistance status; sample size (randomized and completed); sex distribution (male/female); age (mean, SD); rating scale used; depression severity (mean, SD) at baseline, post-treatment, and follow-up; concomitant medication status and any washout period; stimulated brain region (no restriction); stimulation frequency (Hz); intensity as % of motor threshold (MT%); pulses per session and number of sessions; total pulses (calculated when not explicitly reported as pulses-per-session × sessions); and treatment duration.

Outcomes were extracted at post-treatment and follow-up, prioritizing the 2-week post-treatment timepoint. Endpoints comprised continuous depressive-symptom severity and the binary outcomes of response and remission. Where necessary, missing dispersion metrics (e.g., SD) were derived from SEs, CIs, or p values using standard formulae. For cross-over studies, only pre-cross-over data were extracted; multi-arm trials were extracted by arm. We preferentially used intention-to-treat denominators for response/remission when available; otherwise, available-case data were extracted as reported.

### Quality assessment

Risk of bias was assessed using the Cochrane Risk of Bias tool [17]. Two reviewers independently rated each domain as low, unclear, or high risk; disagreements were resolved by consensus or third-party adjudication. An overall risk-of-bias judgment was then assigned for each study.

### Statistical analyses

TMS dose was defined a priori across four dimensions: total pulses, pulses per session, number of sessions, and treatment duration in weeks. For the clinical interpretation of dose-response curves, we pre-specified categorical dose levels (low, mid-range, high) for each dimension, based on established therapeutic ranges from previous studies [7, 8]. Mid-range dose was defined as follows: 30,000–40,000 total pulses; 1,500–2,000 pulses/session; 10–20 sessions; and 2–3 weeks’ duration. Doses below and above these ranges were classified as low and high, respectively. Each dimension was analyzed separately. Treatment effects were summarized as standardized mean differences (SMD) for continuous depressive-symptom scores and log risk ratios (logRR) for binary endpoints (response, remission), with 95% confidence intervals (CIs). For binary data, logRRs and standard errors were computed from 2×2 tables; when any cell count was zero, the Haldane-Anscombe 0.5 continuity correction was applied [18]. Binary outcomes were modeled on the log-risk-ratio scale and are reported as RRs (by exponentiation) with 95% CIs.

Dose-response relations were estimated using a one-stage random-effects model with the between-study variance estimated by restricted maximum likelihood (REML), implemented in *dosresmeta* [19]. Non-linearity was accommodated with restricted cubic splines (three knots at the 10th, 50th, and 90th centiles of the observed dose distribution) [20]. Wald χ^2^ tests evaluated the overall association and departure from linearity. The maximum effective dose (EDmax), defined as the dose associated with the highest predicted benefit within the observed range, was estimated from the fitted spline. Pointwise 95% confidence intervals were obtained using the delta method.

We prespecified a tiered approach for sensitivity analysis. When both the overall dose-response and the non-linearity test were significant, we assessed robustness of the EDmax with leave-one-out re-fits of the spline model, recording interior peaks only [19, 21]. When the overall association was significant but non-linearity was not, we focused on the linear dose trend: a one-stage random-effects meta-regression [22, 23]. We summarized leave-one-out slopes (median, interquartile range, sign reversals, and loss of significance) and compared the linear and spline specifications using the Akaike Information Criterion and a Wald test for non-linearity. Publication bias and small-study effects were examined using contour-enhanced funnel plots and dose-adjusted Egger-type meta-regressions [24]. Two-sided p < 0.05 was considered statistically significant. Analyses were conducted in R (version 4.4.3).

## Results

### Study characteristics

**Figure 1:**
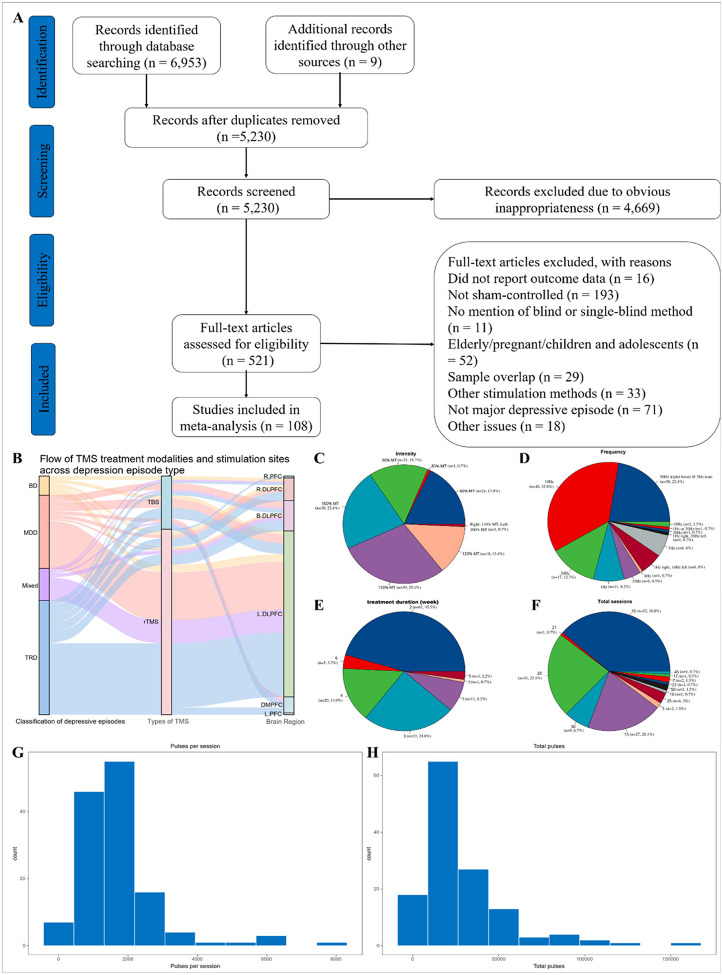
Overview of study characteristics and stimulation parameter distributions among 108 included randomized sham-controlled trials. Panel A Diagram of the preferred reporting items for systematic review and meta-analysis (PRISMA). B shows the flow of TMS treatment modalities and stimulation sites across different types of depressive episodes. Panels C–F summarize stimulation intensity, frequency, treatment duration, and total number of sessions. Panels G–H display the distributions of pulses per session and total pulses across all included trials. BD, bipolar disorder; MDD, major depressive disorder; TRD, treatment-resistant depression; TBS, theta-burst stimulation; rTMS, repetitive transcranial magnetic stimulation; R.PFC, right prefrontal cortex; L.PFC, left prefrontal cortex; R.DLPFC, right dorsolateral prefrontal cortex; L.DLPFC, left dorsolateral prefrontal cortex; DMPFC, dorsomedial prefrontal cortex.

We retrieved 6,962 published records in all three literature databases, of which 108 RCTs with sham arms met inclusion criteria, comprising 134 intervention arms (Fig. 1A) [25–132]. In total, 5,621 participants were enrolled: 3,021 randomized to active rTMS (mean age 43.3 years; 59.2% female) and 2,590 to sham (mean age 42.5 years; 55.8% female). Among the trials, 38 focused on MDD [25, 29, 41, 45, 46, 49–53, 64, 67, 69, 71, 77, 79, 80, 82, 84, 90–92, 96, 97, 101, 103, 110, 111, 114, 118, 119, 121–123, 125, 126, 129, 130], 12 on bipolar disorder (BD) [30, 32, 37, 48, 56, 68, 72, 75, 85, 105, 106, 117], 42 on TRD [26, 28, 31, 33, 35–39, 42, 44, 54, 57–63, 66, 70, 73, 74, 76, 78, 83, 86, 87, 93, 94, 98, 99, 102, 104, 107–109, 112, 113, 116, 128, 131, 132], and 16 included mixed MDD and BD populations [27, 34, 40, 43, 47, 55, 65, 81, 88, 89, 95, 100, 115, 120, 124, 127]. Full study characteristics and demographics are presented in Supplementary **Table S1**.

Across the 134 active arms, 104 used conventional rTMS [25–36, 40–45, 47–50, 53–55, 57–62, 64–67, 69, 71–74, 76–81, 83–87, 89–91, 94, 96–105, 107, 108, 112–125, 128–132] and 30 applied TBS (iTBS and cTBS) [37–39, 45, 46, 50–52, 56, 57, 63, 68, 70, 75, 82, 88, 90, 92, 93, 95, 106, 109–111, 113, 119, 126, 127]. Most targeted the left dorsolateral prefrontal cortex (DLPFC; Fig. 1B, **69**.4%) [25–28, 31–33, 36–40, 42–45, 47, 49, 53, 54, 57–59, 62, 64–74, 77–85, 89–91, 94, 96–105, 107, 109–125, 128–130]. The most frequent parameters were 110% of resting motor threshold (MT; Fig. 1C, **29**.1%) [26, 30, 32, 35, 36, 39, 41–45, 47–50, 54–56, 73, 74, 76, 78, 85–87, 107, 108, 110, 111, 115–117, 122, 129–131], 10 Hz frequency (Fig. 1D, 35.8%) [26, 28, 32, 33, 35, 42–45, 53, 54, 57, 58, 62, 64, 67, 69, 71–74, 77, 80, 81, 84, 90, 91, 96, 97, 99, 101–103, 105, 107, 113–115, 117, 119, 121, 122, 124, 132], 2-week treatment duration (Fig. 1E, 45.5%) [25, 27, 31, 32, 34, 35, 40, 41, 44, 45, 49, 51–53, 55, 57, 59, 69, 70, 73, 76, 81–83, 85–87, 89, 90, 94, 95, 97, 99, 101, 104, 107, 108, 112, 113, 118, 119, 122, 124, 125], 10 total sessions (Fig. 1F, 38.8%) [25, 27, 31, 32, 40, 41, 44, 45, 49, 51, 53, 55, 57, 59, 69, 70, 76, 81, 83, 85, 87, 89, 94, 95, 99, 101, 104, 107, 108, 112–114, 118, 122, 124, 125], 1,600 pulses per session (Fig. 1G, 17.6%) [26, 27, 40, 44, 45, 47, 53, 55, 57, 81, 85, 107, 114, 122, 128], and 16,000 total pulses (Fig. 1H, 13.9%, ranged widely from 1,200 to 160,000 pulses) [27, 40, 44, 45, 53, 55, 57, 81, 85, 107, 114, 122].

Panel A Diagram of the preferred reporting items for systematic review and meta-analysis (PRISMA). B shows the flow of TMS treatment modalities and stimulation sites across different types of depressive episodes. Panels C–F summarize stimulation intensity, frequency, treatment duration, and total number of sessions. Panels G–H display the distributions of pulses per session and total pulses across all included trials. BD, bipolar disorder; MDD, major depressive disorder; TRD, treatment-resistant depression; TBS, theta-burst stimulation; rTMS, repetitive transcranial magnetic stimulation; R.PFC, right prefrontal cortex; L.PFC, left prefrontal cortex; R.DLPFC, right dorsolateral prefrontal cortex; L.DLPFC, left dorsolateral prefrontal cortex; DMPFC, dorsomedial prefrontal cortex.

### Quality of evidence

Risk-of-bias assessments are summarized in Supplementary **Table S2** and **Figure S1**. Sequence generation was low risk in 65 trials (60.2%) and unclear in 43 (39.8%). Allocation concealment was low in 45 (41.7%), unclear in 60 (55.6%), and high in 3 (2.8%). Blinding of participants and personnel was low in 66 (61.1%) and unclear in 42 (38.9%). Blinding of outcome assessment was low in 103 (95.4%) and unclear in 5 (4.6%). Incomplete outcome data were low in 91 (84.3%), unclear in 11 (10.2%), and high in 6 (5.6%). Selective reporting was low in 104 (96.3%) and unclear in 4 (3.7%). Overall risk of bias was rated low in 54 trials (50.0%), unclear in 45 (41.7%), and high in 9 (8.3%).

### Dose-response meta-analysis for total pulses

**Figure 2:**
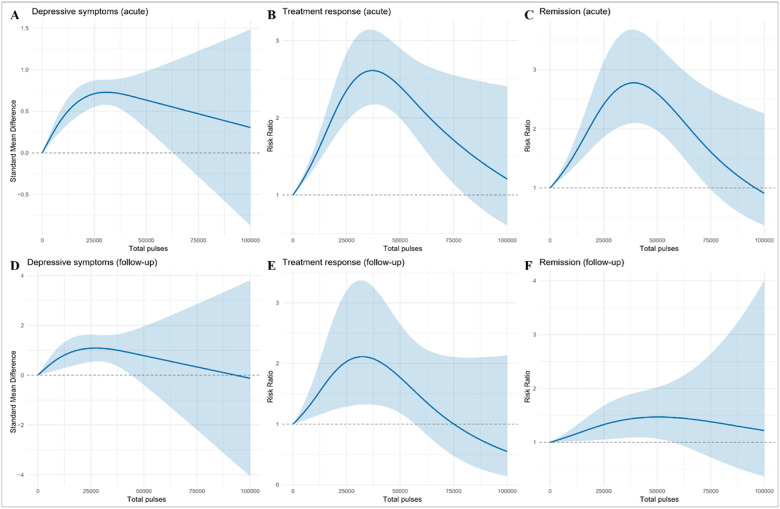
Dose-response associations of rTMS with total pulses across depressive-symptom, treatment-response, and remission outcomes. Panels A, D display standardized mean differences (SMDs) for continuous depressive-symptom scores, and Panels B, C, E, F display risk ratios (RR) for binary endpoints (treatment response and remission), during acute and follow-up periods. Curves were fitted using one-stage random-effects restricted cubic spline models with REML estimation. Shaded areas indicate pointwise 95% confidence intervals.

The dose-response associations for total pulses are shown in Fig. 2 and Supplementary **Table S3**. For depressive symptoms, a significant non-linear pattern emerged. In acute analyses (105 studies, 126 effect sizes), improvement peaked at 30,800 pulses (95% CI 16,100–45,600), with a predicted SMD of 0.73 (95% CI 0.58–0.88), after which the curve plateaued. At follow-up (30 studies; 35 effect sizes), the overall association remained significant but was approximately linear across the observed range, with no evidence of a distinct peak. For treatment response, the acute effect (87 studies, 100 effect sizes) peaked at 37,000 pulses (95% CI 30,700–43,400), with a predicted RR of 2.61 (95% CI 2.18–3.16), and decreased thereafter. At follow-up (14 studies, 16 effect sizes), the optimum was 32,300 pulses (95% CI 25,000–39,700), with a predicted RR of 2.12 (95% CI 1.32–3.35), but effects diminished at higher exposures. For remission, the acute optimum (58 studies, 72 effect sizes) was 39,100 pulses (95% CI 32,000–46,100), with a predicted RR of 2.77 (95% CI 2.10–3.67), followed by gradual decline. At follow-up (11 studies, 11 effect sizes), the curve was flatter, with no clear peak.

### Dose-response meta-analysis for pulses per session

**Figure 3:**
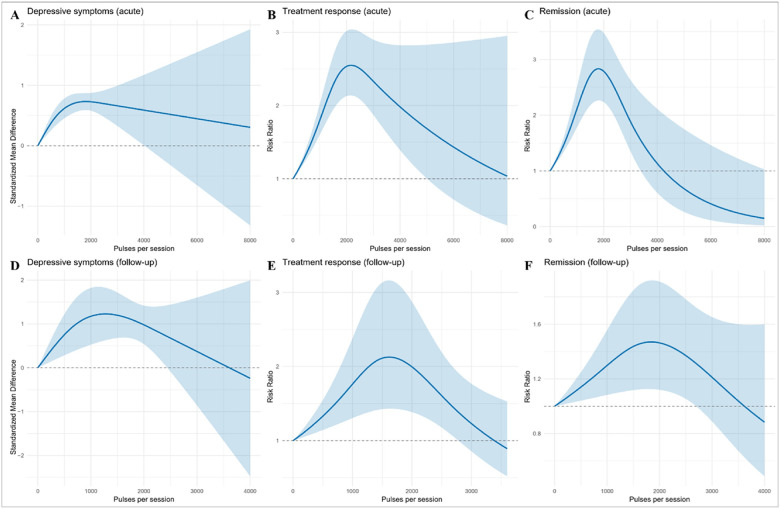
Dose-response associations of rTMS with pulses per session across depressive-symptom, treatment-response, and remission outcomes. Panels A, D display standardized mean differences (SMDs) for continuous depressive-symptom scores, and Panels B, C, E, F display risk ratios (RR) for binary endpoints (treatment response and remission), during acute and follow-up periods. Curves were fitted using one-stage random-effects restricted cubic spline models with REML estimation. Shaded areas indicate pointwise 95% confidence intervals.

Associations between pulses per session and outcomes are shown in Fig. 3 and Supplementary **Table S4**. In acute analyses, depressive symptoms indicated an optimum at 1,800 pulses/session (95% CI 870–2,740), with a predicted SMD of 0.73 (95% CI 0.59–0.87), then gradually declined. At follow-up, the optimum was 1,300 pulses/session (95% CI 870–1,680), with a predicted SMD of 1.23 (95% CI 0.63–1.83), followed by decline. Regarding treatment response, the acute effect peaked at 2,200 pulses/session (95% CI 1,830–2,510), with a predicted RR of 2.56 (95% CI 2.14–3.03), then decreased. At follow-up, the optimum was 1,600 pulses/session (95% CI 1,480–1,760), with a predicted RR of 2.12 (95% CI 1.43–3.16), again followed by decline. Similarly, for remission, the acute peak was 1,800 pulses/session (95% CI 1,610–2,000), with a predicted RR of 2.83 (95% CI 2.27–3.56), after which the effect tapered. At follow-up, the maximum was 1,840 pulses/session (95% CI 1,490–2,190), with a predicted RR of 1.48 (95% CI 1.13–1.92).

### Dose-response meta-analysis for total sessions

**Figure 4:**
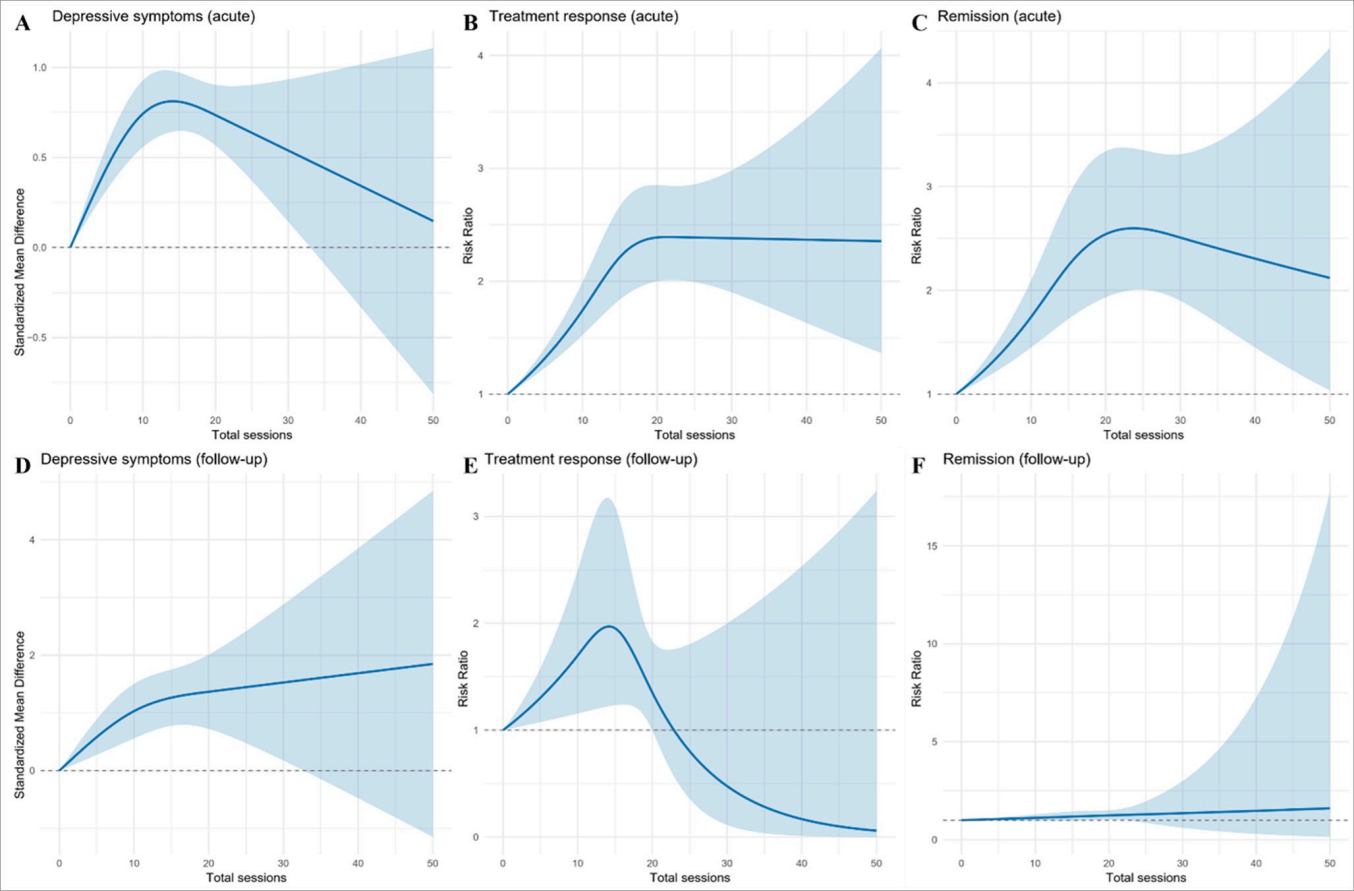
Dose-response associations of rTMS with total sessions across depressive-symptom, treatment-response, and remission outcomes. Panels A, D display standardized mean differences (SMDs) for continuous depressive-symptom scores, and Panels B, C, E, F display risk ratios (RR) for binary endpoints (treatment response and remission), during acute and follow-up periods. Curves were fitted using one-stage random-effects restricted cubic spline models with REML estimation. Shaded areas indicate pointwise 95% confidence intervals.

The associations for total number of sessions are shown in Fig. 4 and Supplementary **Table S5**. In terms of depressive symptoms, acute analyses peaked at 14 sessions (95% CI 11–17), with a predicted SMD of 0.81 (95% CI 0.64–0.98), followed by decline. At follow-up, the association appeared linear across the examined range, with no indication of a distinct peak. For treatment response, the acute optimum was 16 sessions (95% CI 12–19), with a predicted RR of 2.29 (95% CI 1.90–2.77). At follow-up, the dose–response relation remained broadly linear without evidence of a peak or downturn. Regarding remission, acute effects peaked at 23 sessions (95% CI 17–29), with a predicted RR of 2.59 (95% CI 1.99–3.39), followed by gradual decline. At follow-up, no clear maximum was identified; the overall association was not statistically significant (χ^2^=5.09, df = 2; p = 0.078).

### Dose-response meta-analysis for treatment duration

**Figure 5:**
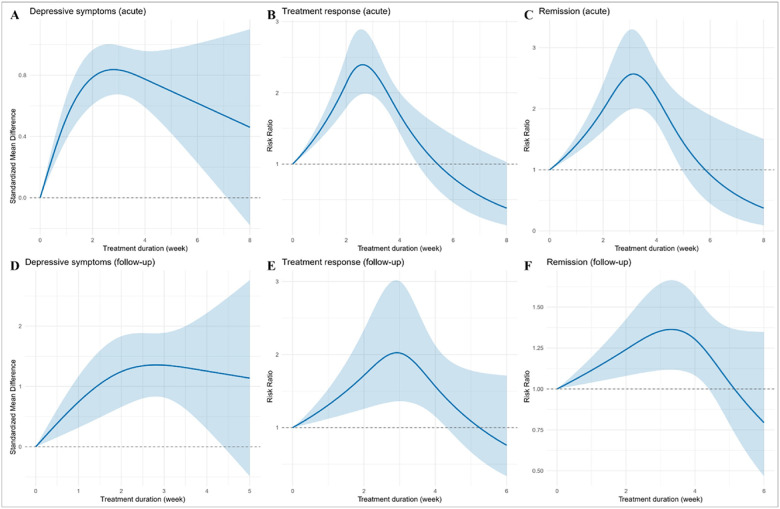
Dose-response associations of rTMS withtreatment duration (week) across depressive-symptom, treatment-response, and remission outcomes. Panels A, D display standardized mean differences (SMDs) for continuous depressive-symptom scores, and Panels B, C, E, F display risk ratios (RR) for binary endpoints (treatment response and remission), during acute and follow-up periods. Curves were fitted using one-stage random-effects restricted cubic spline models with REML estimation. Shaded areas indicate pointwise 95% confidence intervals.

The associations between treatment duration and outcomes are shown in Fig. 5 and Supplementary **Table S6**. Analysis of acute depressive symptoms revealed peak occurred at 2.9 weeks (95% CI 2.4–3.5), with a predicted SMD of 0.84 (95% CI 0.67–1.00). At follow-up, the test for non-linearity was not significant. Acute treatment response rose to a peak at 2.7 weeks (95% CI 2.2–3.2; predicted RR 2.39, 95% CI 1.99–2.89). This outcome at follow-up was maximized at 2.9 weeks (95% CI 2.4–3.5; predicted RR 2.03, 95% CI 1.36–3.00), after which it declined. Finally, the optimum for acute remission was 3.1 weeks (95% CI 2.5–3.7; predicted RR 2.56, 95% CI 2.01–3.32), while the follow-up maximum was 3.3 weeks (95% CI 2.6–4.0; predicted RR 1.36, 95% CI 1.12–1.66), with no further gains beyond this point.

### Subgroup analysis

Eight prespecified subgroup models were examined (rTMS and TBS each assessed for total pulses, pulses per session, total sessions, and treatment duration). For conventional rTMS, acute outcomes consistently demonstrated non-linear associations with mid-range optima: 30,000 to 40,000 total pulses, 1,500 to 2,300 pulses per session, 15 to 17 sessions, and about 3 weeks of treatment. At follow-up, effects were weaker and largely linear; where peaks appeared, they were small and exploratory. For TBS, acute outcomes showed similar patterns but at lower dose levels, with optima around 2,000 pulses per session, 8 to 10 sessions, and 2 to 2.5 weeks of treatment. Total pulse analyses suggested peaks near 40,000 for response, but follow-up evidence was inconsistent, with sparse data (≤ 5 studies in several strata) yielding unstable curves and wide uncertainty.

Taken together, the subgroup analyses indicate that moderate dosing regimens, rather than the highest exposures, are associated with the greatest acute benefit for both rTMS and TBS. Follow-up effects were smaller, often linear, and less precisely estimated, especially for TBS. We therefore highlight acute non-linearity and mid-range plateaus as the most reproducible findings, while treating apparent follow-up peaks as hypothesis-generating given limited data. Full subgroup outputs are reported in Supplementary **Tables S7**–**S14** and **Figs. S2**–**S9**.

### Sensitivity analyses and publication bias

The robustness of our dose-response findings was supported by leave-one-out sensitivity analyses, which demonstrated stable EDmax estimates and linear slopes without sign reversals. Publication bias, evaluated using Egger’s test, was generally absent. Significant small-study effects were observed only in a few outcomes, particularly those assessing follow-up efficacy. Comprehensive results for these sensitivity and publication bias analyses are presented in the Supplementary material (see the Sensitivity analysis section), including detailed summaries (**Table S15**) and supporting visualizations (leave-one-out analyses in **Figures S10**–**S13**; funnel plots in **Figures S14**–**S17**).

## Discussion

In this dose-response meta-analysis of 108 randomized, sham-controlled clinical trials, four rTMS dosing parameters (total pulses, pulses per session, number of sessions, and treatment duration) showed consistent non-linear associations with acute outcomes in MDE [7, 8, 10]. Across continuous and binary endpoints, benefits generally peaked at mid-range exposures and then plateaued or declined, indicating that more stimulation is not invariably better. For conventional rTMS, the dose ranges associated with the highest predicted acute benefit were 30,000–40,000 total pulses, 1,500–2,300 pulses per session, 15–17 sessions, and approximately 3 weeks of treatment. Follow-up effects were smaller, often near-linear, and imprecisely estimated. TBS showed a qualitatively similar pattern, with optima at lower exposures of approximately 2 000 pulses per session, 8–10 sessions, and about 2–2.5 weeks of treatment. However, the evidence base was relatively sparse, particularly for follow-up outcomes.

A key observation is that estimated “best” doses differ across depressive-symptom change (continuous), response (≥ 50% reduction), and remission (scale-defined thresholds). This divergence is expected and clinically informative for three reasons. First, the endpoints capture progressively stricter clinical goals: continuous scores detect early, broad improvements; response requires surpassing a relative threshold; remission requires crossing an absolute low-symptom boundary. As targets become more stringent, curves tend to shift rightward or flatten because additional “consolidation” of benefit is needed to convert partial improvement into threshold-crossing events. Second, the endpoints are modeled on different statistical scales with distinct variance structures and inherent saturation: continuous outcomes summarize mean change on an approximately linear scale, whereas response and remission are estimated on the log-risk scale for Bernoulli outcomes with an implicit sigmoidal link. Even when underlying symptom improvement rises smoothly with increasing dose, probability curves derived from binary outcomes tend to flatten and saturate as they approach their 0–1 limits. This produces different apparent slopes and internal peaks compared with standardized mean differences, reflecting differences in modeling scale rather than in subgroup composition. Third, the four dosing parameters correspond to different phases of clinical change. Pulses per session primarily represent within-session induction, whereas the number of sessions and overall duration reflect between-session consolidation.

Total pulses combine these components and can be achieved through heterogeneous schedules with distinct neuroplastic and tolerability profiles. As a result, each endpoint-specific EDmax represents a different perspective on the same underlying dose–response curve. Because many confidence intervals overlap, these differences should be viewed as indicative rather than prescriptive.

These findings have clear clinical relevance. When the primary goal is to reduce symptoms, treatment regimens set at the moderate dose ranges identified above achieve most of the acute benefit with good efficiency [133, 134]. Achieving remission depends more on adequate consolidation through sufficient sessions and treatment duration, but gains diminish at higher exposures. This pattern is consistent with metaplastic counter-regulation [135] and with practical limits on adherence and tolerability observed in real-world settings [136, 137]. The modest and largely linear associations observed at follow-up suggest that sustaining treatment effects may require additional strategies beyond dose escalation. These include continuation or maintenance TMS [138, 139], optimization of pharmacotherapy [140], and psychotherapy [141] tailored to individual risk profiles and early treatment response.

Beyond these clinical implications, our findings build on and help reconcile previous meta-analyses. Earlier studies reported bell-shaped or plateauing dose–response patterns when total pulses were used to quantify exposure, with indications of a clinical plateau around 3–4 weeks and possible moderation by stimulation frequency [7]. By modelling the four dosing parameters separately and evaluating both acute and follow-up outcomes across continuous and binary measures, our analysis shows that the most consistent pattern is a mid-range plateau during the acute phase. The estimated maxima for total exposure and treatment duration align with findings from total-pulse–based and trajectory analyses [7, 8], but also suggest that increasing exposure beyond typical clinical regimens offers little additional benefit and, in some models, even a decline in efficacy. Several mechanisms may underlie these plateaus. rTMS involves neural plasticity mechanisms regulated by homeostatic or metaplastic processes [142], and excessive or prolonged stimulation can trigger counter-regulatory responses that reduce overall benefit [143]. Clinically, higher stimulation doses can lead to greater fatigue and scalp discomfort, which may limit tolerability. Higher dropout rates have been reported in TMS programmes and can, in turn, reduce the effective dose delivered and overall adherence [144]. Greater pulses per session also tend to co-vary with other parameters, such as inter-train interval, frequency, and intensity, which may alter the balance between facilitatory and inhibitory effects [145]. Although these dose-response analyses cannot fully separate such co-variations, the consistent shape of the curves across endpoints argues against a purely statistical explanation.

Several limitations should be acknowledged. Follow-up data, particularly for TBS, were limited, resulting in wide uncertainty at the extremes of the dose distribution. Although one-stage random-effects spline models help reduce ecological bias and capture non-linear trends, each dose parameter was analyzed separately. Residual confounding by unmeasured factors such as stimulation frequency, target site, intensity, coil type, or navigation method therefore remains possible, and total pulses are mathematically dependent on the other components [146]. Moreover, potential interactions among dose dimensions, including total pulses, pulses per session, number of sessions, and treatment duration, were not directly modeled [147]. Specific combinations of these parameters may exert synergistic or antagonistic influences on treatment outcomes (e.g., depressive-symptom change, treatment response, or remission) [147], which should be further explored in future research. In addition, most included trials targeted the left DLPFC using 10 Hz stimulation at about 110% of the motor threshold for 2–3 weeks of treatment. Generalizability to other stimulation targets (e.g., dorsomedial or right prefrontal cortex) remains limited, and target-specific efficacy differences may exist [66]. Future research could employ network meta-analysis to systematically compare and rank target sites [5]. Finally, variation in sham procedures, concomitant treatments, definitions of treatment-resistant depression, and rating scales also contributes to heterogeneity across studies [148].

In summary, rTMS efficacy increases sharply at lower exposures, reaches its greatest effect at moderate doses, and then levels off or declines. The dose–response maxima vary across endpoints, reflecting differences in clinical definition, statistical scale, and the relative roles of induction and consolidation. These findings support a dosing approach focused on well-calibrated, mid-range regimens that are matched to the therapeutic goal, such as symptom improvement, treatment response, or remission, and highlight the importance of maintenance strategies to sustain benefit over time.

## Supplementary Files

This is a list of supplementary files associated with this preprint. Click to download.

- Supplementarymaterials.docx
- PRISMAChecklist.doc

## Data Availability

The data used in this meta-analysis were extracted from previously published studies cited in the reference list. The data extraction and analytical code are available from the corresponding author upon reasonable request.
